# Retrospective, Longitudinal, One-Group Study on the Implementation of Continuous Glucose Monitoring To Improve Quality of Care for Patients With Type I or II Diabetes Mellitus in an Internal Medicine Residency Continuity Community Clinic

**DOI:** 10.7759/cureus.64594

**Published:** 2024-07-15

**Authors:** Andrey Manov, Rakahn Haddadin, Sukhjinder Chauhan, Elizabeth Benge

**Affiliations:** 1 Internal Medicine, MountainView Hospital, Las Vegas, USA

**Keywords:** diabetes mellitus type 2, diabetes mellitus type 1, hypoglycemia, glucose variability, glucose management indicator (gmi), hba1c, continuous glucose monitoring (cgm)

## Abstract

In this three-year retrospective study, data from 51 patients with type 1 or type 2 diabetes mellitus (DM), receiving a minimum of 3-4 insulin injections per day and self-monitoring their blood glucose (SMBG) four times a day, were derived from our internal medicine residency primary care clinic. The patients were equipped with a continuous glucose monitoring (CGM) device that shared 24-hour glucose data with the clinic. They were assigned to members of our CGM team, which included internal medicine or transitional year medical residents who functioned under the supervision of a board-certified endocrinologist. The residents, in consultation with our endocrinologist, assessed the patients' glucose management data and adjusted their treatment regimens biweekly by calling the patients, and monthly by seeing the patients in the clinic.

Significant results from the study include a reduction in HbA1c from 9.9% to 7.6%, an average blood glucose decrement from 242 mg/dL to 169 mg/dL, a reduction in the incidence of mild hypoglycemia from below 70 mg/dL to 54 mg/dL, from 4.68% to 0.76% per day, and a more pronounced hypoglycemia with glucose less than 54 mg/dL from 3.1% per day to 0.2% per day. We observed a significant increase in the time in the range of the blood glucose from 33% to 67% per day. Furthermore, 9.5% of the patients in this study eventually discontinued their daily insulin injections and continued treatment with oral diabetic medications with or without the use of injectable GLP-1 receptors once a week.

Our study affirms that CGM devices significantly improve glycemic control compared to SMBG, supporting its efficacy in optimizing glycemic control in real-world clinical practice. The results imply that this can be accomplished in internal medicine residency clinics and not exclusively in specialized endocrine clinics. As far as we know, this is the first study of its kind in a residency clinic in the USA.

This study confirms the benefits of widening the application of CGM in DM, along with the challenges that must be overcome to realize the evidence-based benefits of this technology. CGM needs to become a part of routine monitoring for type 1 and type 2 DM.

## Introduction

In the United States, diabetes is very prevalent, with 90 million individuals living with impaired glucose tolerance or impaired fasting blood glucose (BG), often referred to as pre-diabetes. Approximately 35 million Americans have diabetes mellitus (DM) [1]. Diabetes is the seventh leading cause of death in the USA. The complications stemming from DM are multifactorial. The major causes of death among patients with DM are macrovascular diseases like major cardiovascular events (MACE), stroke, and congestive heart failure. DM also causes microvascular complications. The number one cause of blindness in the US is diabetic retinopathy, and end-stage renal disease due to diabetic nephropathy is the leading cause of renal replacement therapy in the world. Diabetic neuropathy is also very prevalent in patients with DM. The management of DM requires multidisciplinary measures. The linchpin of effective diabetes management lies in achieving and maintaining optimal BG control, thereby decreasing both immediate and long-term complications. In our evolving understanding of diabetes management, monitoring tools have played a pivotal role. These tools, which range from the more traditional self-monitoring blood glucose (SMBG) meters 3 to 4 times a day to the advanced continuous glucose monitoring (CGM) systems, have allowed us to gain a more nuanced comprehension of the strengths and limitations of glucose control as stated in the Standards of Care for Diabetes in 2020 [2, 3, 4, 5]. Monitoring BG 3-4 times a day is important for achieving good diabetic control, especially for patients reliant on insulin treatment, such as those with T1DM and T2DM on multiple injections of insulin per day. Its limitations in providing a comprehensive profile of BG fluctuations have spurred a quest for more effective solutions [4, 5, 6]. In this pursuit, CGM has emerged as a game-changer, affording individuals a comprehensive and detailed overview of their BG levels throughout 24 hours and not just at one point in time like with SMBG. Furthermore, the ability to share data with healthcare providers has transformed diabetes management by enabling providers to track patterns and trends in BG levels, facilitating timely and effective adjustments tailored to individual needs. Additionally, CGM throughout the day has encouraged patients, including those who previously struggled with adherence, to become more accountable for their dietary habits, medication adherence, and physical activity [4,5,6,7]. In this initiative, we harnessed the potential of CGM devices compared to SMBG to improve the management of patients with uncontrolled diabetes who were using 3-4 injections of insulin per day [7]. This retrospective study was conducted within an internal medicine clinic, with the primary participation of the medicine residents. The project was supervised by an endocrinologist. The primary objective of this endeavor was to illuminate the effectiveness of CGM devices in improving the management of uncontrolled DM in our internal medicine residency clinic compared to SMBG in patients with DM using multiple daily injections of insulin (MDII). The most important parameters using CGM are time in range (TIR) with BG between 70-180 mg/dl and the goal, as per the American Diabetes Association (ADA), above 70% of the 24 hours, above 17 hours per day. The goal as per the ADA for mild hypoglycemia (less than 70 mg/dl) and more pronounced hypoglycemia is less than 4% for the former and less than 1% for the latter, respectively. The targets using CGM as per ADA are high BG between 180 and 250 mg/dl, less than 25%, and very high BG above 250 mg/dl, less than 5%. Our project was designed to comprehensively evaluate the impact of CGM device integration into our internal medicine residency clinic's protocols. Our goal was to analyze retrospectively a range of critical glycemic control parameters, including the glucose management indicator (GMI) which approximates HbA1c levels, average BG levels, the incidence of hypoglycemic events, and the duration of TIR where target blood sugar ranged between 70-180 mg/dl. By systematically assessing these metrics, we intended to gain deeper insights into the benefits and efficacy of CGM technology in enhancing the care provided to patients with uncontrolled DM in a medicine residency clinic. We know that many studies have shown the superiority of the CGM compared to SMBG in specialized endocrine clinics. The goal was to achieve better glycemic control in the population of the sickest patients with DM in a project primarily managed by medicine residents. We also believe that this improves the quality of education for our medical residents. This retrospective study represents a step toward optimizing diabetes management within our clinical setting, and we hope our findings will contribute to the broader understanding of how CGM devices can be implemented in other internal medicine residency clinics to improve patient outcomes and the education of internal medicine physicians.

## Materials and methods

A retrospective three-year study was conducted on 51 adult patients between the ages of 21 and 90 with uncontrolled DM in the internal medicine residency primary care clinic. The main question in this retrospective study was whether it was possible to successfully introduce CGM by internal medicine and transitional year residents under the supervision of board-certified endocrinologists and improve diabetes control by monitoring the CGM data and intervening based on them. This question is important because it shows that improvement in diabetic management can be achieved by switching from SMBG to CGM in internal medicine clinics, and not only in specialized endocrine clinics, in a process governed by residents. Patients were monitored by the CGM team as the usual standard of practice. Our CGM team included internal medicine and transitional year medical residents, as well as a board-certified endocrinologist who was a member of the clinic. The patients were sampled based on the inclusion and exclusion criteria for uncontrolled T1DM or T2DM (Table 1). Of the patients, 17% had T1DM and 83% had T2DM. Forty-three percent of patients were females and 57% were males. At the time of switching from SMBG to CGM, all patients were using 3-4 injections of insulin per day in addition to oral antidiabetic medications or GLP1-RAG. All subjects were current patients at the residency clinic and were given information regarding CGM as a standard practice. The decision to use CGM was based on patients' wishes and insurance coverage. The goal of the study was to show improvement of the GMI and TIR, as well as a decrease in mild and more pronounced hypoglycemia, after switching the patients from SMBG to CGM under the supervision of the CGM team. The study results were obtained retrospectively for the past 3 years. The inclusion and exclusion criteria are displayed in Table 1.

**Table 1:** Inclusion and exclusion criteria. CGM: Continuous Glucose Monitoring; SMBG: Self-monitoring blood glucose.

Inclusion Criteria	Exclusion Criteria
Participants ages 18-80 years old	Participants not following dietary and lifestyle recommendations
Participants with the diagnosis of type 1 or type 2 diabetes mellitus	Participants who do not understand the utilization of the CGM
Participants with HbA1c > 7% and who were receiving their primary care in the Internal Medicine residency Clinic in Nevada	Participants wearing their CGM device less than 70% of the time
Participants with uncontrolled blood glucose levels while using SMBG for equal or greater than four times daily	Participants with cognitive problems
Participants receive their primary care only at the Internal Medicine Residency Clinic in Nevada	Participants not showing in the clinic for more than two visits
Participants can use a CGM device	Participants who are pregnant or incarcerated
Patients on 3-4 injections of insulin +/- oral diabetic medications	Patients unresponsive to calls from the clinic
The patient can adjust their insulin based on the CGM data	Patients whose insurance did not cover the CGM device

In the clinic, study participants were given written instructions on how to use CGM and treat their diabetes based on the data received. They were counseled about their diet and exercise and provided a pamphlet containing different foods, meals, carbohydrates, and caloric content. Patients were required to show an understanding of the procedure and teach back this understanding to the CGM team members before the CGM implementation. This is a standard procedure before starting CGM. The CGM Dexcom G6 and G7 devices were used in the study because they were available, did not require calibration, and because of the availability of technical team support. Data were collected from the CGM Dexcom database through the Dexcom Clarity database and the clinic EHR. Patients with device-compatible iPhones or Android phones were given a share code by the CGM team so that the CGM team could monitor their BG data continuously and adjust the diabetes treatment. Patients without compatible phones were given a receiver to monitor their BG. Patients were seen at the clinic once a month. The patients' data were downloaded from their receiver at the clinic visit, and their insulin regimen could be adjusted appropriately. The patients in our study were also contacted by a member of the CGM team every two weeks, and their insulin regimens were adjusted based on their BG readings with the help of our board-certified endocrinologist as a standard of care for patients with CGM.

Data collection

Data from the CGM database were used, and the data were shared with the clinic if the patients had compatible phones. For those who did not have such phones, the data were obtained by talking to the patients on the phone and during their clinical visits from their CGM receiver. The data analyzed are described in Table 2.

**Table 2:** Data collected and evaluated. SMBG: Self-monitoring blood glucose; GMI: Glucose Management Indicator.

Variable	Operational Definition
HbA1c (GMI)	HbA1c was measured as a percentage of erythrocytes bound with glucose.
Average blood glucose	The average blood glucose was measured in milligrams per deciliter while using SMBG.
Time in range (TIR)	TIR was measured as a percentage of glucose between 70-180 mg/dl throghout the 24-hour period.
Mild hypoglycemia	Mild hypoglycemia was measured by percent of the blood glucose between 69-54 mg/dl in a 24-hour period.
Pronounced hypoglycemia	More pronounced hypoglycemia was measured by the percent of the blood glucose below 54 mg/dl in a 24-hour period.

Statistical analysis

The statistical methods used were the five paired t-tests. A Bonferroni adjustment was used to achieve the alpha value at 0.01. The differences in HbA1c, TIR, average BG, and percent spent in mild and more pronounced hypoglycemia were assessed. The difference scores for A1C, TIR, and average BG were normally distributed, as assessed by Shapiro-Wilk's test (p = 0.200; p = 0.200; p = 0.074, respectively) and as evaluated by a normal Q-Q Plot. However, difference scores for the percent of time spent in mild hypoglycemia and in pronounced hypoglycemia violated the assumption of normality, as assessed by Shapiro-Wilk's test (p < 0.001; p < 0.001, respectively). As a result, the differences in A1C, TIR, and average BG were analyzed via a paired t-test. The differences in the percent of time spent in mild hypoglycemia and in pronounced hypoglycemia were analyzed via the nonparametric equivalent, the Wilcoxon signed-rank test.

To use the Wilcoxon signed-rank test to determine if there is a statistically significant median difference between two related groups, the shape of the distribution of the differences needs to be symmetrical. The difference scores for both the percent of time spent in mild hypoglycemia and in pronounced hypoglycemia were approximately symmetrically distributed, as assessed by a histogram with a superimposed normal curve. Consequently, the difference in scores for these variables was analyzed via the Wilcoxon signed-rank test.

## Results

Seventeen percent of our patients had T1DM and 83% had T2DM. Fifty-seven percent were males and 43% were females. A total of 9.5% of our patients discontinued their insulin due to improved diabetic control and continued their treatment with oral diabetic medications, with or without GLP1-Receptor agonists (Table 3).

**Table 3:** Descriptions of patients with DM included in our retrospective study. DM: Diabetes mellitus; CGM: Continuous glucose monitoring.

	Percentage of patients
Type 1	17%
Type 2	83%
Male	57%
Female	43%
Discontinued insulin after CGM	9.5%

The mean HbA1c before starting the CGM in the patients was 9.9%, TIR was 33%, average BG was 242 mg/dl, mild hypoglycemia between 69 and 54 mg/dl was 4.68% or 1 hour and 19 minutes per day, and lower than 54 mg/dl was 3.1% or 32 minutes per day. The descriptive statistics of measured variables before and after CGM introductions are described in Table 4.

**Table 4:** Descriptive statistics of measured variables. TIR: Time in range; BG: Blood glucose.

Variable	Median	Mean	SD
PreA1c	9.20	9.914	2.217
PostA1c	7.40	7.625	1.340
PreTIR	31.00	0.336	0.207
PostTIR	67.00	0.669	0.202
PreAvgBG	230.00	242.255	65.485
PostAvgBG	167.00	169.431	34.581
PreMildHypo	5.00	0.047	0.006
PostMildHypo	0.00	0.008	0.014
PreProHypo	3.10	0.031	0.013
PostProHypo	0.00	0.002	0.006

**Figure 1:**
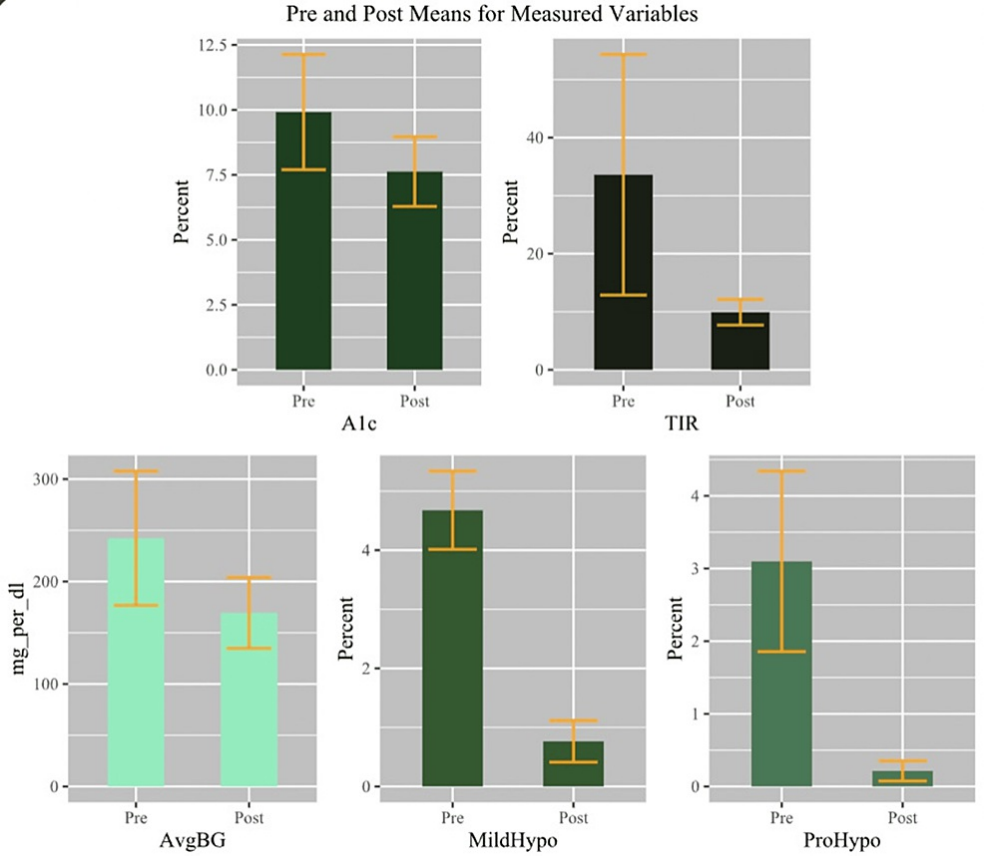
Bar graphs displaying each measured variable with standard error bars.

The paired samples t-test revealed the mean difference was significantly higher for the TIR, indicating patients spent a greater amount of time in the target range (33.33, 95% CI (44.505, 22.216), t(50) = 5.993, p < 0.001, d = 0.839). The Wilcoxon signed-rank test showed (Table 4; Figure 1) a significant median difference in the percentage of mild hypoglycemia (Mdn = -3.1, z = -6.193, p < 0.001) and pronounced hypoglycemia (Mdn = -5.0, z = -6.224, p < 0.001). Results indicate that after the introduction of a CGM system, both mild and pronounced hypoglycemia exhibited a median decrease. The results of the paired samples t-test (Figure 1) revealed the mean difference was significantly lower after the introduction of a CGM system in patients’ A1c (-2.288, 95% CI (-1.665, -2.912), t (50)= -7.373, p < 0.001, d = -1.032) and average BG (-72.824, 95% CI (-53.540, -92.107), t(50) = -7.585, p < 0.001, d = -1.062).

There was a decrease in the HbA1c measured by GMI of the CGM by 2.3%. The TIR between 70 and 180 mg/dl increased from 33% before the introduction of CGM to 67% after the introduction of CGM. The average BG decreased after the introduction of the CGM from 242 mg/dl to 169 mg/dl. The incidence of mild hypoglycemia after the introduction of the CGM decreased from 4.68% to 0.76% per day. After the introduction of CGM, the time spent with BG levels less than 54 mg/dl per day decreased from 3.1% to 0.2% per day. All measures were within the ADA goals using CGM [8]. Our patients had a high blood sugar level of 180-250 mg/dl at 27% per day and very high blood sugar at 4% per day after the introduction of the CGM, which was within the ADA goals using CGM and considering that some of our patients were older with a high risk of hypoglycemia [8].

## Discussion

The major advancement in diabetology in the last 20 years was the implementation of CGM. Compared to SMBG, these devices provide a complete picture of the 24-hour glucose values and the ability to act on high or low blood sugar values in real time. SMBG provides BG at one point in time, usually 3-4 times a day, and lacks the complete 24-hour picture of BG values [7]. For patients on multiple injections of insulin per day, using SMBG is like driving in an unknown place without having a map. CGM devices are more user-friendly and are currently covered by more insurance plans. Investigations of these devices in patients with T1DM have indicated that CGM devices significantly reduce patients' HbA1c levels compared to SMBG [8,9]. This new technology will improve the lives of people with diabetes by measuring their glucose every 5 minutes and transmitting the data to their mobile devices. From there, glucose data can be shared with the internal medicine residency clinic, and frequent decisions about treatment can be made. This will allow better glycemic control for patients using this technology.

Different types of clinical studies have shown the advantages of CGM over SMBG in improving diabetes control and reducing hypoglycemia [8-13]. One of the major indications for using CGM is unexpected hypoglycemia in patients on multiple injections of insulin per day, hypoglycemia unawareness, or labile DM. Improvement in hypoglycemia has been observed with the use of CGM in patients with T1DM and T2DM [12, 13]. The Glycemic Management Indicator (GMI) obtained from the CGM is not influenced by conditions such as anemia, chronic kidney disease, race, or cirrhosis of the liver, among others. The current recommendations are to implement CGM for BG control in patients on multiple injections of insulin per day. TIR has become a valuable marker for glucose control, and the goals are to maintain BG between 70 and 180 mg/dl for more than 70% of the day. There are exceptions for older patients, patients at risk of hypoglycemia with more relaxed goals, or pregnant patients with T1DM or T2DM with more stringent goals. CGM devices in different trials have shown a reduction in HbA1c levels by 0.5%.

These devices also reduced hypoglycemia in insulin-requiring DM patients [13, 14]. CGM devices play a pivotal role in enhancing patient engagement and self-management [5]. The availability of continuous glucose data empowers individuals with DM to make informed decisions about their dietary choices, physical activity, and insulin dosing, fostering a proactive approach to managing their condition and improving their quality of life, eating patterns, motivation, and satisfaction with diabetic management compared to SMBG. Additionally, these devices offer alerts and alarms for high and low glucose levels, enabling timely interventions and minimizing the risk of severe hypoglycemic events [11, 15]. However, challenges persist, including the cost of CGM devices and sensors, adherence issues, and technological limitations [16]. Furthermore, the interpretation and utilization of the extensive data provided by CGM devices require adequate patient education and support and are also used as a motivational tool [15, 16]. In our retrospective three-year study, we found that switching from SMBG to CGM led to significant improvements in the health outcomes of the 51 patients with T1DM or T2DM. We observed significant reductions in HbA1c levels, BG levels, hypoglycemic events, and time in range. The average HbA1c reduction was from 9.91% to 7.63%, with a reduction of the mean BG as discussed above and an increase in achieving a TIR to 67%, which is in line with current recommendations. The mild and more pronounced hypoglycemia per day after the switch from SMBG to CGM also decreased significantly as discussed above. Our findings suggest that using CGM instead of SMBG methods can lead to better glycemic control, fewer side effects, and lifestyle improvements. Additionally, 9.5% of our patients with type 2 DM stopped their insulin because of improved glucose control. They were transitioned to oral diabetic agents with or without injectable once-a-week GLP-1 receptor agonists. Our study had its limitations. The sample size was small with 51 patients, the patients' compliance with diet and lifestyle interventions varied, and the follow-up of the patients was up to three years.

Many of the patients who had SMBG before starting the CGM were ill-informed about their diabetic goals, eating requirements, physical activity, and their disease. The study was conducted in one internal medicine residency clinic. Also, six patients had mild anemia of chronic disease, 12 patients had stage 3 and three patients had stage 4 chronic kidney disease, which might have influenced HbA1c levels before starting the CGM. The strength of the study was the implementation of CGM in the internal medicine residency primary care clinic, primarily managed by internal medicine and transitional year residents under the supervision of an endocrinologist. To our knowledge, this is the first implementation in the US in an internal medicine residency clinic of CGM to manage the most difficult-to-treat patients with DM. This implementation not only improved the quality of care for diabetic patients but also enhanced the education of internal medicine residents with the use of modern technical devices in diabetology.

## Conclusions

In this three-year retrospective study, we observed the significance of CGM in managing both T1DM and T2DM compared to SMBG in patients on multiple injections of insulin per day. Providing real-time glucose data and trends, CGM enables patients and physicians to make informed decisions about diet, exercise, and medication regimens, ultimately leading to better health outcomes. This model is excellent and can be implemented by other internal medicine residency programs, potentially improving the education of medical residents. However, randomized prospective trials with larger sample sizes are needed to fully assess the potential of CGM implementation and guided treatment of DM in internal medicine residency primary care clinics.
